# Tuberculosis preventive treatment in people living with HIV—Is the glass half empty or half full?

**DOI:** 10.1371/journal.pmed.1003702

**Published:** 2021-09-14

**Authors:** Olivia Oxlade, Hannah Rochon, Jonathon R. Campbell, Dick Menzies

**Affiliations:** 1 McGill International TB Centre, McGill University, Montreal, Canada; 2 Department of Epidemiology, Biostatistics & Occupational Health, McGill University, Montreal, Canada; 3 Respiratory Epidemiology and Clinical Research Unit, Centre for Outcomes Research & Evaluation, Research Institute of the McGill University Health Centre, Montreal, Canada

## Abstract

Olivia Oxlade and co-authors introduce a Collection on tuberculosis preventive therapy in people with HIV infection.

The most recent World Health Organization Global Tuberculosis (TB) Report suggests that 50% of people living with HIV (PLHIV) newly enrolled in HIV care initiated tuberculosis preventive treatment (TPT) in 2019 [1]. TPT is an essential intervention to prevent TB disease among people infected with *Mycobacterium tuberculosis*—some 25% of the world’s population [2]. Without TPT, it is estimated that up to 10% of individuals will progress to TB disease. Among PLHIV, the prognosis is worse. Of the approximately 1.4 million annual deaths from TB, 200,000 occur among PLHIV [1], who experience TB at rates more than 30 times [3] higher than people living without HIV.

In 2018, governments at the United Nations High-Level Meeting (UNHLM) on TB committed to rapid expansion of testing for TB infection and provision of TPT [4]. The goal was the provision of TPT to at least 24 million household contacts of people with TB disease and 6 million PLHIV between 2018 and 2022. However, by the end of 2019, fewer than half a million household contacts had initiated TPT, well short of the pace needed to achieve the 5-year target [1]. On the other hand, approximately 5.3 million PLHIV have initiated TPT in the past 2 years [1], with particularly dramatic increases in countries supported by the President’s Emergency Plan for AIDS Relief (PEPFAR) [5]. Globally, among PLHIV entering HIV care programs, TPT initiation rose from 36% in 2017 to 49% in 2018 and 50% in 2019 [6,7].

To provide insight into scaling up TPT for PLHIV, it is important to consider each of the many steps involved in the “cascade of care” for TPT. A previous systematic review of studies in several populations receiving TPT concluded that nearly 70% of all people who may benefit from TPT were lost to follow-up at cascade of care steps prior to treatment initiation [8]. To maximize the impact of TPT for TB prevention among PLHIV, the full TPT cascade of care must be assessed to identify problems and develop targeted solutions addressing barriers at each step. Until now, these data had not been synthesized for PLHIV.

In order to address important research gaps related to TPT in PLHIV such as this one, we are now presenting a Collection in *PLOS Medicine* on TPT in PLHIV. In the first paper in this Collection, Bastos and colleagues performed a systematic review and meta-analysis of the TPT cascade of care in 71 cohorts with a total of 94,011 PLHIV [9]. This analysis highlights key steps in the cascade where substantial attrition occurs and identifies individual-level and programmatic barriers and facilitators at each step. In stratified analyses, they found that losses during the TPT cascade were not different in high-income compared to low- or middle-income settings, nor were losses greater in centers performing tests for TB infection (tuberculin skin test [TST] or interferon gamma release assay [IGRA]) prior to TPT initiation.

The net benefits of TPT could potentially be increased through greater adoption of shorter rifamycin-based TPT regimens, for which there is increasing evidence of greater safety, improved treatment completion, and noninferior efficacy, compared to isoniazid regimens. Two reviews of rifamycin-based regimens in mostly HIV–negative adults and children concluded that they were as effective for prevention of TB as longer isoniazid-based regimens, with better treatment completion and fewer adverse events [10,11]. However, safety and tolerability of TPT regimens can differ substantially between people with and without HIV, and for rifamycin-based TPT regimens, safety outcomes were actually worse in people without HIV [12], plus there can be important drug–drug interactions between rifamycin-based regimens and antiretroviral drugs [13]. Reviews of studies focused on PLHIV concluded that TPT (regardless of regimen selected) significantly reduced TB incidence [14] and that the benefits of continuous isoniazid in high TB transmission settings outweighed the risks [15]. As part of this Collection, Yanes-Lane and colleagues conducted a systematic review and network meta-analysis of 16 randomized trials to directly and indirectly compare the risks and benefits of isoniazid and rifamycin-based TPT regimens among PLHIV [16]. Their findings highlight the better safety, improved completion, and evidence of efficacy, particularly reduced mortality, with rifamycin-based TPT regimens, while also noting improved TB prevention with extended duration mono-isoniazid regimens. Their review also revealed that few studies exist on some important at-risk populations, such was pregnant women and those with drug-resistant TB infection.

In North America, recommendations changed in 2020 to favor shorter rifamycin-based regimens over isoniazid [17], but WHO still favors isoniazid [18], largely due to the lower drug costs. Although drug costs for rifamycins are typically higher than for isoniazid, their shorter duration and better safety profile mean that total costs for care (including personnel costs) may be lower for rifamycin-based regimens, even in underresourced settings [19]. The cost-effectiveness of different TPT regimens among PLHIV in underresourced settings remains uncertain, as well as the impact of antiretroviral therapy (ART), and the use of diagnostic tests for TB infection, such as TST or IGRA on cost efficiency. Uppal and colleagues, in the third paper in this Collection, performed a systematic review and meta-analysis of 61 published cost-effectiveness and transmission modeling studies of TPT among PLHIV [20]. In all studies, TPT was consistently cost-effective, if not cost saving, despite wide variation in key input parameters and settings considered.

When comparing access to TPT among PLHIV to household contacts, many would consider the glass is half full, given that almost half of all PLHIV newly accessing care initiated TPT in 2018 and 2019, and the UNHLM goal of 6 million PLHIV initiating TPT was already nearly achieved by the end of 2020. This remarkable achievement is the result of strong recommendations from WHO for TPT among PLHIV for nearly a decade and strong donor support. These policies are, in turn, based on clear and consistent evidence of individual benefits from multiple randomized trials, plus consistent evidence of cost-effectiveness from many economic analyses as summarized in the papers in this Collection. These are useful lessons for scaling up TPT for other target populations, particularly household contacts, of whom less than half a million have initiated TPT, of the 24 million–person target set in 2018.

However, the glass of TPT among PLHIV is also half empty. In contrast to the “90-90-90” targets, 50% of PLHIV newly enrolled in care do not initiate TPT, and PLHIV still bear a disproportionate burden of TB. Programmatic scale-up of TPT continues to encounter challenges that need to be overcome in order to translate individual-level success to population-level improvement. The study by Bastos and colleagues in this Collection has identified programmatic barriers including drug stockouts and suboptimal training for healthcare workers, but it also offers useful solutions, including integration of HIV and TPT services [9]. New evidence on the success of differentiated service delivery will also be invaluable to support programmatic scale-up in different settings [21]. Acting on this evidence will be essential to achieve the goal of full access to effective, safe, and cost-effective TPT for PLHIV.

## Abbreviations

ART: antiretroviral therapy
IGRA: interferon gamma release assay
PEPFAR: President’s Emergency Plan for AIDS Relief
PLHIV: people living with HIV
TB: tuberculosis
TPT: tuberculosis preventive treatment
TST: tuberculin skin test
UNHLM: United Nations High-Level Meeting
